# Predictive quantitative ultrasound radiomic markers associated with treatment response in head and neck cancer

**DOI:** 10.2144/fsoa-2019-0048

**Published:** 2019-11-26

**Authors:** William T Tran, Harini Suraweera, Karina Quaioit, Daniel Cardenas, Kai X Leong, Irene Karam, Ian Poon, Deok Jang, Lakshmanan Sannachi, Mehrdad Gangeh, Sami Tabbarah, Andrew Lagree, Ali Sadeghi-Naini, Gregory J Czarnota

**Affiliations:** 1 Department of Radiation Oncology, Sunnybrook Health Sciences Centre, Toronto M4N 3M5, Canada; 2 Department of Radiation Oncology, University of Toronto, Toronto M5T 1P5, Canada; 3 Evaluative Clinical Sciences Platform, Sunnybrook Research Institute, Toronto M4N 3M5, Canada; 4 Department of Radiotherapy & Oncology, Sheffield Hallam University, Sheffield, UK; 5 Department of Physics, Ryerson University, Toronto M5B 2K3, Canada; 6 Physical Sciences Platform, Sunnybrook Research Institute, Toronto M4N 3M5, Canada; 7 Department of Medical Biophysics, University of Toronto, Toronto M5G 1L7, Canada; 8 Department of Electrical Engineering & Computer Sciences, Lassonde School of Engineering, York University, Toronto M3J 1P3, Canada

**Keywords:** chemoradiation, head and neck carcinoma, predictive assay, quantitative ultrasound, radiation therapy, radiomic

## Abstract

**Aim:**

We aimed to identify quantitative ultrasound (QUS)-radiomic markers to predict radiotherapy response in metastatic lymph nodes of head and neck cancer.

**Materials & methods:**

Node-positive head and neck cancer patients underwent pretreatment QUS imaging of their metastatic lymph nodes. Imaging features were extracted using the QUS spectral form, and second-order texture parameters. Machine-learning classifiers were used for predictive modeling, which included a logistic regression, naive Bayes, and *k*-nearest neighbor classifiers.

**Results:**

There was a statistically significant difference in the pretreatment QUS-radiomic parameters between radiological complete responders versus partial responders (p < 0.05). The univariable model that demonstrated the greatest classification accuracy included: spectral intercept (SI)-contrast (area under the curve = 0.741). Multivariable models were also computed and showed that the SI-contrast + SI-homogeneity demonstrated an area under the curve = 0.870. The three-feature model demonstrated that the spectral slope-correlation + SI-contrast + SI-homogeneity-predicted response with accuracy of 87.5%.

**Conclusion:**

Multivariable QUS-radiomic features of metastatic lymph nodes can predict treatment response *a priori*.

## Lay abstract

In this study, quantitative ultrasound (QUS) and machine-learning classification was used to predict treatment outcomes in head and neck cancer patients. Metastatic lymph nodes in the neck were scanned using conventional frequency ultrasound (US). Quantitative data were collected from the US-radiofrequency signal *a priori*. Machine-learning classification models were computed using QUS features; these included the linear fit parameters of the power spectrum, and second-order texture parameters of the QUS parametric images. Treatment outcomes were measured based on radiological response. Patients were classified into binary groups: radiologic complete response (CR) or radiological partial response (PR), which was assessed 3 months following treatment. Initial results demonstrate high accuracy (%Acc = 87.5%) for predicting radiological response. The results of this study suggest that QUS can be used to predict head and neck cancer response to radiotherapy *a priori*.

Each year, 550,000 head and neck (H&N) cancer cases are diagnosed globally and comprises tumors of the pharynx, larynx, oral cavity, nasal cavity, paranasal sinuses, and salivary glands [1]. Most H&N tumors arise from epithelial cells; thus, squamous cell carcinoma (SCC) accounts for approximately 90% of cases. The risk factors associated with H&N cancer include tobacco use, alcohol consumption, and human papilloma virus (HPV) infection [2]. There is also evidence to suggest that Epstein–Barr virus is implicated in H&N oncogenesis [3], as well as hereditary factors, and diseases such as Fanconi anemia [4]. Genetic drivers of H&N cancer have been linked to mutations in the *p53* gene, and deregulation of the PI3K/Akt/mTOR pathway, which are responsible for regulating cell cycling, survival, and differentiation [2].

Distant metastasis at the time of presentation is rare and accounts for only 10% of patients. Therefore, locoregional treatments are often required to treat the primary tumor and regional lymph nodes [1]. Treatments for H&N cancer are dependent on the initial staging and may involve surgery, radiotherapy (XRT) or systemic therapy. XRT is administered as a curative treatment, or as adjuvant therapy, with or without concurrent chemotherapy (i.e., chemoradiation) depending on the clinical presentation [1]. However, the primary intent is to achieve locoregional control (LRC) with long-term disease-free survival [5]. It is estimated that 75% of patients with SCC of the H&N will derive a benefit from XRT in terms of achieving LRC [6]. Therefore, XRT is frequently used up-front for curative intent and as part of a multimodality treatment plan [6].

The standard XRT dose is 70 Gy/35 fractions to the gross tumor volume (GTV) and affected lymph nodes, although different fractionation schemes are also practiced [7,8]. Intensity modulated radiation therapy (IMRT) is now a technical mainstay and uses inverse planning to achieve steep dose drop-off gradients that result in superior dose conformality around the target site with minimal dose to normal surrounding tissues [5]. Also, IMRT plans have benefited greatly from image-guided XRT to correct for anatomical displacements during each treatment fraction, resulting in superior target localization. Despite these technical and treatment advances, there remains a clinical problem for a subset of patients who do not respond to treatment; Peng *et al.* showed that the 5-year mortality rate was approximately 20% despite patients having received IMRT treatment [9]. Another report indicated that the annual mortality rates confer 75,000 deaths overall from oral cavity, pharyngeal, and laryngeal malignancies combined [10]. Taken together, local relapse and death from treatment failure is still a clinical challenge.

To address the clinical challenges associated with aggressive tumors that demonstrate treatment resistance, response-guided and adaptive XRT have been the focus of intense research. Radiomics-based markers from several modalities have been investigated recently to measure intratreatment tumor response. The field of radiomics in oncologic imaging includes characterizing pixel or voxel attributes and their relative spatial relationships within the image layout. Radiomic data can yield high-dimensional imaging markers and information about tumor biology (i.e., the imaging phenotype); for example, characterizing hypoxia, intratumor heterogeneity, tissue structure, and biochemical composition [11–13]. In the context of radiomics-guided therapy, it may provide an opportunity to adapt and tailor radiation treatments based on physiological information extracted from quantitative imaging either before or during treatment [14,15].

In previous studies, predictive (*a priori*) and early-response quantitative ultrasound (QUS) radiomic markers have demonstrated promising results; for example, measuring breast tumor response to chemotherapy. Sadeghi *et al.* reported significant changes in the acoustic backscatter signal as early as 4 weeks after the start of chemotherapy [16]. Recent studies have indicated that texture analyses of QUS-radiomic features can be carried out with multivariate machine-learning algorithms to predict treatment responses with high accuracy [17,18]. These studies have demonstrated that QUS-radiomic markers are capable of characterizing tumors in terms of structural properties (i.e., tissue heterogeneity) and functional attributes; demonstrating a significant correlation to treatment response outcomes.

Here, we report a novel application of QUS spectroscopy to predict treatment response in H&N cancer. Quantitative radiomic features derived from QUS are computed from the radiofrequency (RF) backscatter signals that are typically discarded after B-mode image reconstruction. However, in QUS the RF data is retained and analyzed by applying a Fourier transform to the acoustic backscatter intensity signal, followed by regression analysis of the frequency-dependent power spectrum. The QUS-radiomic parameters that are determined using this technique include, the mid-band fit (MBF), 0-MHz intercept (special intercept [SI]) and spectral slope (SS) [19–22]. Other QUS-radiomic features are also obtained by estimates of the backscatter coefficient, which relate to tissue microstructure properties (i.e., size and concentration of scatterers) [20,23]. Compared with other imaging modalities such as positron emission tomography (PET) and MRI, QUS imaging requires only relatively inexpensive ultrasound (US) technology and does not require contrast agents. Additionally, the major advantages of QUS compared with conventional gray-scale sonography include independence from user and machine settings. In this study, we apply these principles within the framework of H&N XRT. Pretreatment, QUS-derived radiomic features were obtained from the transformed image of metastatic lymph nodes of 32 H&N cancer patients and we report radiomic signatures obtained *a priori* that were associated with XRT treatment response endpoints.

## Materials & methods

### Patients & treatment

This study was approved by the institutional research ethics board. There were 32 subjects enrolled in this study. Informed written consent was obtained from all participants. All subjects had a biopsy confirmed diagnosis of H&N cancer.

As part of the patients’ standard of care, histological analyses of the primary tumor were performed: including HPV and Epstein–Barr virus [24–26] assays. The patients’ diagnostic workup also included a pretreatment computed tomography (CT) and MRI scan to obtain radiological information about the initial tumor size and extent (i.e., primary tumor size, lymph node status, lymph node size, and tumor histological features).

All patients in this study were given chemoradiation or definitive radiation alone (70 Gy over 33 fractions). An expansion of 5 mm on the GTV was created to form the high-dose clinical target volume on the primary and nodal volume (70 Gy). Another expansion margin of 1 cm was added to the GTV to create the CTV56 volume. Elective nodal areas were treated to a dose of 56 Gy. All XRT was administered using modern IMRT or VMAT techniques [8,27]. A summary of the concomitant systemic therapies used and, radiation treatments are outlined in Table 1. Patients were selected to ensure neck nodes were sufficiently large and accessible for imaging purposes.

**Table 1.** Demographic and clinical information.

Patients characteristics	n (%)
**Patient and clinical characteristics n = 32 (all subjects)**	
Age (median)	60 years old
Sex: – Male – Female	29 (90.1) 3 (9.4)
**Tumor and node status**	
Primary tumor (T): – T1 – T2 – T3 – T4	2 (6.3) 23 (71.9) 4 (12.5) 3 (9.4)
Node involvement (N): – N1 – N2 (all sub-classifications) – N3	10 (31.3) 20 (62.5) 2 (6.3)
Histological type: – Squamous cell carcinoma – (+) nonkeratinizing nasopharyngeal carcinoma – Small cell carcinoma	30 (93.8) 1 (3.1) 1 (3.1)
HPV status: – Indeterminate – p16(+) – p16(-)	9 (28.1) 22 (68.8) 1 (3.1)
Pretreatment lymph node size (mean, cm ± SD)	
Complete responders	2.35 ± 0.66
Partial responders	2.71 ± 0.65
**Treatment characteristics**	
Chemotherapy + radiation (concomitant): – Cisplatin (high dose) – Cisplatin (low dose) – Cisplatin → Carboplatin – Carboplatin – Carboplatin + Etoposide	27 (84.4) 23 (71.8) 1 (3.1) 1 (3.1) 1 (3.1) 1 (3.1)
Targeted therapy + radiation (concomitant): – Cetuximab	1 (3.1) 1 (3.1)
Definitive radiation alone	4 (12.5)
**Post-treatment (3-month assessment from magnetic resonance imaging)**	
Complete response (locoregional control)	13 (40.6)
Partial response (locoregional failure)	19 (59.4)

EBV+: Epstein–Barr virus positive carcinoma; p16+/-: Human papilloma virus (HPV) status (positive/negative); SD: Standard deviation.

### Treatment response evaluation

Treatment response was evaluated from clinical follow-up with radiological assessment using dynamic contrast enhanced MRI 3 months after completing treatments. Patients were categorized according to their clinical response into binary classes as either complete responders or partial responders. Patients who were complete responders demonstrated LRC after XRT, in other words, no residual disease at the primary site or within lymph nodes. In contrast, patients were classified as partial responders if there was residual disease at the primary site (and/or the lymph nodes) after the 3-month assessment. Patients who had residual disease after (chemo)radiation were seen for surgical consideration and salvage.

### US data acquisition

The largest lymph node was identified with the guidance of the patient's treating oncologist. A ‘baseline’ US scan of the largest involved lymph node was completed for each patient, 2 weeks prior to starting (chemo)radiation, at the time of CT-planning for XRT.

US data included grayscale (b-mode) images and the digitized RF signal. Data were collected from a commercially available US device that was customized to retain the RF data (Elekta Ltd, CA, USA). A linear 4D transducer (4DL14-5/38 Linear 4D, BK Ultrasound, MA, USA) was used for imaging, which had a center frequency of approximately 8 MHz and a sampling rate of 40 MHz. Data were acquired across the entire lymph node volume, along 256 lateral scan lines (in-plane; 3.8 cm lateral field of view), with a maximum axial depth of 5 cm. The acoustic focus was adjusted for the depth of the lymph node for each patient (average depth = 2.5 cm).

### QUS feature extraction

QUS analyses were completed using individual RF lines in-plane, within a region-of-interest that was selected across the entire target lymph node. A sliding window technique was employed with a window block of 2 × 2 mm and 94% overlap between the adjacent windows in both axial and lateral directions. In order to remove effects of system transfer functions and beam forming by the transducer, normalization of the spectral form was carried out by calibrating the signal to the spectra of a tissue-mimicking phantom, with known acoustic properties [28]. Imaging and measurements were conducted by trained members of a clinical research team. Previous work from the same research group shows that QUS acquisition by different sonographers and different systems do not significantly affect the data [29].

An attenuation correction was calculated based on the point-compensation method [30] by estimating the local attenuation coefficient [31]. There were six QUS parameters computed from the power spectra which included: MBF, SS, SI, spacing among scatterer (SAS), average scatterer diameter (ASD), and average acoustic concentration (AAC).

The MBF, SS, and SI parameters were derived by applying a linear regression over the normalized power spectrum within a -6 dB window [32]. The ASD and average acoustic concentration parameters were derived by fitting a spherical Gaussian model, as well as a fluid-filled sphere model (Anderson model) to the backscatter coefficient [33,34]. The SAS was derived by applying an autoregressive model on to the power spectrum and using Burg's recursive algorithm [35]. For SAS only, determination power spectrum normalization was performed using a planar reflector reference phantom. The SAS was determined by computing the autocorrelation of the normalized power spectrum and the frequency at which the peak occurred during the autocorrelation [36–38]. A parametric map was generated for every QUS-derived radiomic feature.

Texture analysis was completed on QUS parametric maps using a gray-level co-occurrence matrix (GLCM) algorithm [39]. The GLCM features were calculated from neighboring pixel relationships, based on the relative pixel-to-pixel distances and gray-level tones [39,40]. Each GLCM was composed of 16 gray-tone values and was constructed at four inter-pixel distances, within four spatial directions (0, 45, 90, and 135°). Four textural features were extracted from each GLCM (Equations 1–4) and were subsequently averaged over all GLCMs for each parametric image. The four textural features extracted and found to be most useful were contrast (con), correlation (cor), energy (ene), and homogeneity (hom).

(Eq. 1) $$Contrast\;=\;\sum_{i=1}^{N_{g}}\sum_{j=1}^{N_{g}}{\left(i-j\right)}^{2}\;p(i,j)$$

(Eq. 2) $$Correlation\;=\frac{\sum_{i=1}^{N_{g}}\sum_{j=1}^{N_{g}}\left(i\right)(j)p(i,\;j)-\mu_{x}\mu_{y}}{\sigma_{x}\sigma_{y}}$$

(Eq. 3) $$Energy=\sum_{i=1}^{N_{g}}\sum_{j=1}^{N_{g}}{\left(p(i,\;j)\right)}^{2}$$

(Eq. 4) $$Homogeneity=\sum_{i=1}^{N_{g}}\sum_{j=1}^{N_{g}}\frac{p(i,\;j)}{1+{\left(i-j\right)}^{2}}$$

For the texture computations, *p*(*i,j*) is the probability of having two neighbor pixels with gray-level intensities of *i* and *j* in the map, while *Ng* is the number of quantized gray-level intensities. The mean and standard deviations are symbolized as *μ* and *σ*, respectively and were calculated for both *i^th^* rows and *j^th^* columns of the matrix. Contrast describes the degree of pixel-neighbor contrast probabilities in the image, while correlation describes the linear dependency between neighboring pixel pairs. The energy measures textural uniformity within neighboring pixels and homogeneity measures the incidence of pixel pairs at different intensities [39]. A total of 41 radiomic features were extracted from the QUS data, including spectral parameters and four image texture features calculated from each spectral parametric map.

### Statistical analysis

Each QUS mean value and QUS-texture parameter was averaged over the entire lymph node mass. A Shapiro–Wilk test was used to test for normality. The differences of means between complete response (CR) and partial response (PR) groups was determined by either an independent sample t-test for normally distributed data (two-sided, 95% CI), or a Mann–Whitney U test (two-sided, 95% CI) for datasets that did not exhibit a normal distribution. The statistical tests were performed on SPSS V.22 (IBM Corporation, NY, USA). A significance level of less than 0.05 was considered significant.

Machine-learning classifiers, including a *k*-nearest neighbor (*k*-NN) and a naive-Bayes were used to classify partial versus complete responders using the QUS parameters. A leave-one-out cross-validation (LOOCV) scheme at subject level was used to evaluate the performance of the two classifiers. The left-out patient (test sample) in each iteration remained unseen during classifier parameter tuning (such as *k = 1*, in *k*-NN classifier), and feature selection. Since there were 41 features and only 32 samples, the feature selection within a given model was limited based on the curse of dimensionality; wherein the number of selected features must be less than the number of samples in the training set divided by a factor of ten in order to mitigate overfitting [41]. Therefore, a maximum of three features were selected by the classification models using a sequential forward selection model in a wrapper framework [42] and by executing a cross-validation on the training set at each fold of the leave-one-out evaluation scheme. The feature selection method learns which features are most informative at each iteration, choosing the next features based on already selected features and the internal constraints of the classifier. In order to avoid the peaking phenomenon due to the curse of dimensionality, ten samples per class were required for each feature (i.e., 1/10 of the sample set), given there were only 13 samples in the CR class, the best single feature was selected and used for evaluating the performance of the classifiers. Selection was continued to a maximum of three best features for this sample population. For each radiomic model, the sensitivity (%S_n_), specificity (%S_p_), accuracy (%Acc), and area under the curve (AUC) of the receiver-operating characteristic (ROC) were calculated as the criteria for the performance of the classifiers. The AUC was calculated based on the prediction for each test sample in the leave-one-out cross validation, then pooled and calculated over the entire dataset. CIs were calculated and reported for predictive models. Machine-learning classification, as well as QUS feature extraction was conducted on MATLAB-based software (Mathworks, MA, USA). Statistical analysis was conducted for CR (*n*_1_.#x00A0;= 13) and PR (*n*_2_.#x00A0;= 19).

## Results

### Patients & tumor characteristics

Participants in this study had a median age of 60 years (range = 40–82 years old); 29 subjects were male and 3 were female. There were 13 patients who demonstrated a CR, whereas 19 patients demonstrated a PR. Among the SCC patients, 22 patients had HPV-positive tumors, one patient had an HPV-negative tumor and seven patients had indeterminate results. Table 1 summarizes the clinical characteristics of participants.

The mean pretreatment lymph node size was 2.35 ± 0.66 cm for complete responders and 2.71 ± 0.65 cm for partial responders, which were superficial in depth (i.e., <5 cm from surface) for US examination. Histological H&N tumor types were primarily SCC (93.8%), Epstein–Barr virus positive nonkeratinizing undifferentiated nasopharyngeal carcinoma (3.1%), and small cell carcinoma (3.1%). The primary tumor sites included the oral cavity, oropharynx, hypopharynx, nasopharynx, glottic larynx, supraglottic larynx, and parotid gland.

### Treatment details

Treatment characteristics are outlined in Table 1. Concomitant high-dose cisplatin chemotherapy as a radiation sensitizing agent was given to 23 patients (100 mg/m^2^; q. 21 days). One patient received low-dose weekly cisplatin (40 mg/m^2^; q.1wk) + concomitant XRT. One patient was started on high-dose cisplatin, but later switched to carboplatin due to severe neuropathy; one patient received two of the three prescribed doses (2/3) of carboplatin (70 mg/m^2^/day IV on days 1–4, 22–25 and at days 43–46 of radiation) + concomitant XRT but did not complete the third cycle of chemotherapy, due to chemotherapy-related toxicity. One patient with small cell carcinoma received carboplatin AUC 5 IV on day 1; Etoposide 100 mg/m^2^ IV days 1–3 for q. 21 days + concomitant XRT. At last, one patient was prescribed cetuximab (initial loading dose of 400 mg/m^2^ followed by weekly administration of 250 mg/m^2^ for 7 weeks) + concomitant XRT. Four patients in the cohort received XRT alone using altered fractionation with twice-daily radiation, once a week. All patients received 70 Gy in 33 fractions.

### QUS-radiomic measurements

**Figure 1.**
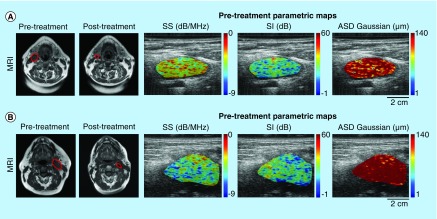
Lymph node imaging. (Left panel) representative of T1-weighted MR and (right panel) contoured B-mode ultrasound images with QUS parametric map overlays of SS, SI, and ASD Gaussian for a complete responder **(A)** and a partial responder **(B)** pre to post treatment. The MR images have been contoured to delineate the lymph node that was scanned. The displayed QUS-GLCM parameters demonstrated significant differences between complete responder and partial responder groups in texture analysis. Ultrasound image dimensions are: axial distance (image height) 4 cm, lateral distance (horizontal length) 6 cm. Scale bar = 2 cm (ultrasound image). ASD: Average scatterer diameter; GLCM: Gray-level co-occurrence matrix; MRI: Magnetic resonance imaging; QUS: Quantitative ultrasound; SI: Special intercept; SS: Spectral slope.

Representative magnetic resonance, US B-mode images, and pretreatment lymph node parametric maps are presented in Figure 1. Figure 1A presents data from a representative patient determined clinically to be a complete responder. In the clinical MRI data (left panel) there is a diminishment in nodal size to a clinically insufficient size. Figure 1B presents data from a partial responder with minimal nodal change. Panel sets (A) and (B) also presents representative QUS parametric images for SS, SI, and ASD (acoustic scatter diameter determined using a Gaussian fit). As is evident, there are visible differences in the pretreatment parametric images between the complete responder and partial responder patients with obvious changes in the mean values of parameters.

**Figure 2.**
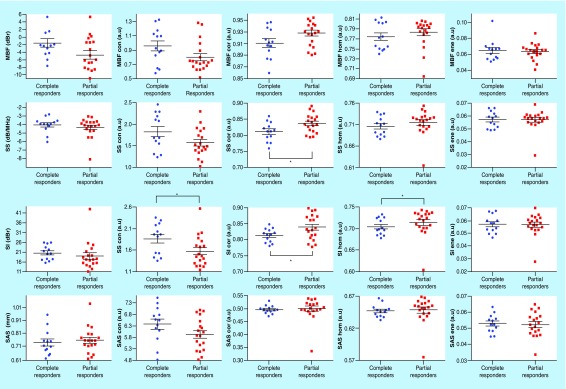

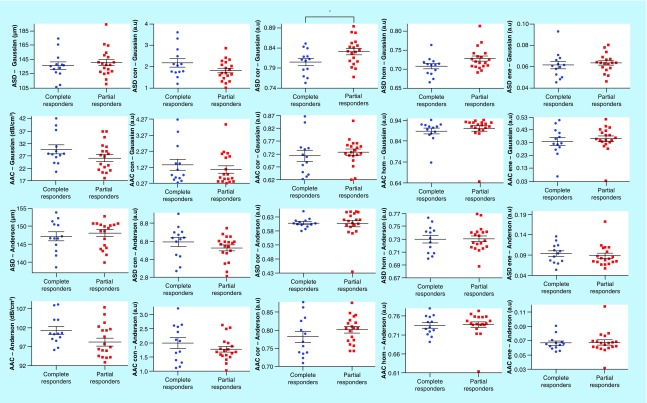
Scatter plots of the quantitative ultrasound mean-value and textural parameters for complete responders and partial responders at pretreatment. Error bars represent ± one standard error of the mean. *p < 0.05. AAC: Average acoustic concentration; ASD: Average scatterer diameter; SAS: Spacing among scatterer; SI: Special intercept; SS: Spectral slope.

Figure 2 presents representative data for all QUS-radiomic parameters between complete responders versus partial responders. This includes parameters which were determined to be statistically, significantly different and parameters which demonstrated no statistically significant differences between patients that were complete responders versus partial responders. This data corresponds to Table 2, which presents the significant and near-significant QUS-mean-values and QUS-textural parameters for the two response groups (i.e., CR vs PR). The mean QUS parameters did not demonstrate a statistically significant difference between complete responders and partial responders. However, the MBF demonstrated near significance; the mean MBF value was -1.49 ± 4.06 dB for complete responders versus -4.70 ± 4.63 dB for partial responders (p = 0.052). Textural analyses demonstrated both near significant and significant differences between response groups. Near significant features included the MBF-cor and the SS-con (p = 0.059 and p = 0.061, respectively). Five QUS-GLCM parameters demonstrated a statistically significant difference between groups: SS-correlation (p = 0.018), SI-contrast (p = 0.022), SI-correlation (p = 0.018), SI-homogeneity (p = 0.044), and ASD Gaussian correlation (p = 0.031). Overall, the texture-based features demonstrate greater sensitivity and specificity to detect differences between the response groups (Table 2). The texture-based parameters that were most effective were related to the contrast and correlation of specific ultrasound-based parameters.

**Table 2.** Mean quantitative ultrasound values and mean quantitative ultrasound gray-level co-occurrence matrix feature values for complete responders and partial responders.

	Parameter (Units)	Mean value (CR)	Mean value (PR)	p-value
MBF	(dB)	-1.49 ± 4.06	-4.70 ± 4.63	0.052
MBF-cor	(AU)	0.91 ± 0.26	0.93 ± 0.02	0.059
SS-con	(AU)	1.84 ± 0.43	1.57 ± 0.33	0.061
**SS-cor**	**(AU)**	**0.81 ± 0.03**	**0.84 ± 0.03**	**0.018**
**SI-con**	**(AU)**	**1.86 ± 0.36**	**1.57 ± 0.36**	**0.022**
**SI-cor**	**(AU)**	**0.81 ± 0.02**	**0.84 ± 0.04**	**0.018**
**SI-hom**	**(AU)**	**0.70 ± 0.02**	**0.71 ± 0.03**	**0.044**
**ASD (gau)-cor**	**(AU)**	**0.80 ± 0.03**	**0.83 ± 0.03**	**0.031**
AAC (and)	(dB/cm^3^)	101.23 ± 3.78	98.19 ± 4.58	0.058

Reported values are mean ± one standard error of the mean. Bold parameters demonstrate statistical significance between groups. Other features approached near significance.

AAC: Average acoustic concentration; ASD: Average scatterer diameter; AU: Arbitrary unit; CR: Complete response; dB: Decibel; MBF: Mid-band fit; PR: Partial response; QUS: Quantitative ultrasound; SI: Special intercept; SS: Spectral slope.

In order to understand which parameters were best for the *a priori* prediction of treatment response, machine-learning approaches were undertaken to evaluate the predictive capabilities of single and multiple parameters in combination. Classification methods were compared and specifically ROC were computed to evaluate the performance of QUS parameter(s) using a logistic regression classifier, naive-Bayes classifier, and then a *k*-NN approach. Table 3 presents results using a logistic regression classifier for univariate, 2-feature, and 3-feature models. The univariate feature sensitivity ranged between 61 and 69% and specificity ranged between 63 and 68%. Univariate results showed that the SI-cor demonstrated the lowest predictive performance with a sensitivity of 61.5% and a specificity of 63.2% (AUC = 0.682). In contrast, the best univariate feature using the logistic regression classification method was the SI-con, which demonstrated a sensitivity of 61.5%, specificity of 68.4% and AUC = 0.741. Logistic regression analyses for combination features showed that the combination using three features, in other words, SS-correlation + SI-contrast + SI-homogeneity, resulted in the highest classification performance (S_n_ = 85%, S_p_ = 84% and AUC = 0.91).

**Table 3.** Results for the best univariate (A), bivariate (B) and multivariate (C) features using a logistic regression classifier.

Predictive QUS radiomic models from univariate and multivariate feature sets				
**A: Univariate feature model**	**%S_n_**	**%S_p_**	**AUC**	
– SS-cor	69.2	68.4	0.737	
**– SI-con**	**61.5**	**68.4**	**0.741**	
– SI-cor	61.5	63.2	0.682	
– SI-hom	61.5	63.2	0.713	
– ASD (gau)-cor	61.5	63.2	0.704	
– MBF	61.5	63.2	0.688	
**B: 2-feature multivariate model**	**%S_n_**	**%S_p_**	**AUC**	**%Acc**
**– SI-con + SI-hom**	**76.9**	**78.9**	**0.870**	**84.4**
– SI-con + ASD (gau)-cor	69.2	68.4	0.740	71.9
**C: 3-feature multivariate model**	**%S_n_**	**%S_p_**	**AUC**	**%Acc**
– SI-con + SI-hom + MBF	76.9	78.9	0.879	84.4
**– SS-cor + SI-con + SI-hom**	**84.6**	**84.2**	**0.911**	**87.5**

Bold fields represent the best performing feature within the feature set.

Acc: Accuracy; ASD: Average scatterer diameter; AUC: Area under the curve; MBF: Mid-band fit; QUS: Quantitative ultrasound; SI: Special intercept; Sn: Sensitivity; Sp: Specificity; SS: Spectral slope.

**Figure 3.**
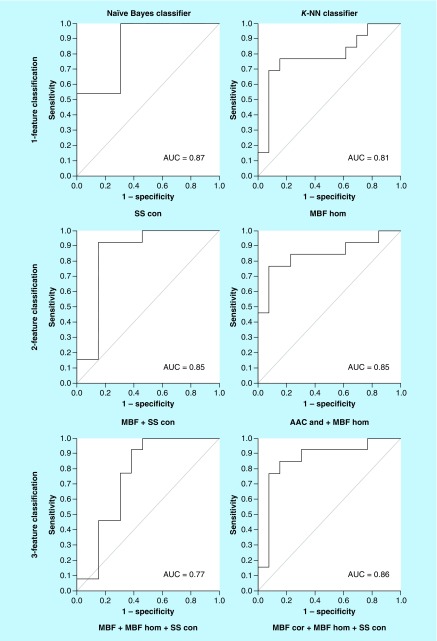
Results of univariable, two-feature and three-feature classification models using naive Bayes and *k*-nearest neighbor classifiers. Models with the greatest AUC values are presented. AAC: Average acoustic concentration; AUC: Area under the curve; *k*-NN: k-nearest neighbor; MBF: Mid-band fit; PR: Partial response; SS: Spectral slope.

**Figure 4.**
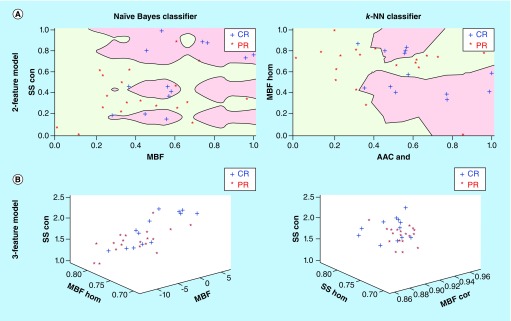
Multivariable feature spaces for datasets using naive Bayes and *k*-nearest neighbor classifiers. **(A)** Two-feature classification and decision boundaries (green and pink regions) are presented for CR (+) or PR (*) data samples within the feature space. **(B)** Representative three-feature classification showing the relative spatial boundaries between CR versus PR samples. Feature axes have been normalized. AAC: Average acoustic concentration; CR: Complete response; *k*-NN: k-nearest neighbor; MBF: Mid-band fit; PR: Partial response; SS: Spectral slope.

Table 4 presents the results of cross-validated classifications of complete and partial responders in advance of treatment using the naive-Bayes and *k*-NN classifiers for a single feature, two features, and three features. The numbers below the AUC in parentheses show the 95% CI. The corresponding ROC curves for naive-Bayes and *k*-NN classifiers for one, two, and three features are presented in Figure 3. Naive-Bayes classification performed best for univariate and two-feature set; whereas, k-NN classification was better for classifying CR/PR using a three-feature model. For a single-feature model, an accuracy of 91.5% was achieved by the SS-contrast (%S_n_ = 85.8%, %S_p_ = 97.3% and AUC = 0.866) using the naive-Bayes classifier for baseline data. Using two QUS and texture features (i.e., MBF+ SS-con), the classification performance showed a sensitivity of 80.4%, a specificity of 93.9% and an AUC of 0.848 (%Acc = 87%). The *k*-NN classifier for three features had an accuracy of 83.3%, which corresponded to an AUC of 0.859 using the following parameters: MBF-cor + MBF-hom + SS-con. Figure 4 presents the naive-Bayes and *k*-NN feature space for QUS parameters wherein the respective axes are the selected features of that model with each sample distributed in space. The red and green areas of the two-feature classification are the decision boundaries for response. The *k*-NN analyses for univariate feature sets corresponded with an accuracy of 77.1% (MBF homogeneity parameter; S_n_ = 71%, %S_p_ = 83.5% and AUC = 0.810). Also, the *k*-NN classifier performed better as the number of features was increased from one to two features; the %S_n_, %S_p_ and AUC for two features were 78.5%, 83.5% and 0.845, respectively. All classification values for naive-Bayes and *k*-NN classification models are summarized in Table 4.

**Table 4.** Results for the best univariable (A), bivariable (B) and multivariable (C) features with machine-learning algorithms, *k*-nearest neighbor and naive-Bayes.

Machine learning classification using QUS radiomic features					
**A: Univariate models**					
**Classifier**	**%S_n_**	**%S_p_**	**AUC (95% CI)**	**%Acc**	**Univariate Model**
– naive-Bayes	85.8	97.3	0.866 (0.73,1.01)	91.5^†^	SS-con
*– k*-NN	71.0	83.5	0.810 (0.64, 0.98)	77.1	MBF-hom
**B: 2-feature classification**					
**Classifier**	**%S_n_**	**%S_p_**	**AUC (95% CI)**	**%Acc**	**2-feature multivariable model**
– naive-Bayes	80.4	93.9	0.848 (0.70, 1.00)	87.1^†^	MBF + SS-con
*– k*-NN	78.5	83.5	0.845 (0.69, 1.00)	81.0	AAC (and) + MBF-hom
**C: 3-feature classification**					
**Classifier**	**%S_n_**	**%S_p_**	**AUC (95% CI)**	**%Acc**	**3-feature multivariable model**
– naive-Bayes	72.3	84.6	0.766 (0.59, 0.94)	78.5^†^	MBF + MBF-hom + SS-con
*– k*-NN	77.7	88.9	0.859 (0.71, 1.00)	83.3	MBF-cor + MBF-hom + SS-con

† Note that the decrease in classification performance using the naive Bayes classifier from univariate to multivariable models is due to a peaking phenomenon. For this model, the best classification was based on a univariate feature set.

AAC: Average acoustic concentration; %Acc: Accuracy percentage; AUC: Area under the curve; *k*-NN: *k*-nearest neighbor; MBF: Mid-band fit; QUS: Quantitative ultrasound; Sn: Sensitivity; Sp: Specificity; SS: Spectral slope.

## Discussion

In this study, we report that the pretreatment QUS-radiomic signatures obtained in metastatic H&N lymph nodes taken *a priori* are predictive of clinical treatment endpoints. This first clinical report for QUS in H&N cancers demonstrates that using both uniparameter and multiparametric QUS-radiomic markers from texture analyses can be used to differentiate complete responders from partial responders up-front. There are significant differences in the texture-based parameters related to SS, SI, and ASD parametric images. The results of this study suggest that metastatic lymph nodes demonstrate imaging phenotypes that correspond to the treatment responses of primary H&N cancer tumors.

Radiomic markers for treatment response in H&N cancer have been studied previously; for example, PET [14,15,43]. Lehtiö *et al.* reported that the pretreatment tumor blood flow parameters from PET imaging were associated with response outcomes [43]. In that study, high blood flow markers were linked to poor local control after XRT [43]; while subjects that demonstrated a higher fractional hypoxic volume also had worse survival outcomes (p = 0.036) [43]. More recent studies using PET to measure pretreatment and early hypoxia in HPV+ H&N cancer have been useful for biomarker-guided XRT [15]. Other studies have investigated quantitative MRI to monitor XRT response; Cao *et al.* reported techniques in dynamic contrast enhanced MRI to measure alterations in tumor blood volume (BV) and blood flow before and after 2 weeks of starting XRT [44]. The results showed a significant increase in the BV for patients who had locally controlled disease; whereas, patients who had local failure had an insignificant change in the BV [44].

Other MRI techniques, such as diffusion-weighted MRI measure the apparent diffusion coefficient; a study by King *et al.* purported a significantly lower apparent diffusion coefficient, as measured after 2 weeks of XRT, for patients who had local treatment failure [45]. Another use of radiomics in H&N cancer was also to differentiate between benign and malignant lymph nodes, and to characterize cervical lymphadenopathy using dynamic susceptibility contrast MRI [46,47]. Indeed, these studies represent a significant effort to identify markers at early time intervals, since early detection can potentially inform response-guided XRT, avoid ineffective treatment regimens, and avert unnecessary side effects. Two recent studies in H&N cancer also used machine-learning to predict clinical outcome. Valliėres *et al.* used radiomic features from the PET-CT data and clinical features of 300 H&N cancer patients to predict locoregional recurrence and distant metastases with an AUC of 0.69 and 0.86, respectively [48]. Leger *et al.* compared the performance of 11 machine-learning algorithms and 12 feature selection methods to find an optimal prediction model of locoregional tumor control using CT data [49]. Although these previous studies hold great promise, the major limitations for MRI- and PET-CT based radiomic tests include high equipment and imaging costs, limited portability of these systems and radiation exposure, as is the case for PET imaging. QUS is poised to address some of these limitations, with the capability of probing tissue microstructure and characterizing malignancies based on their acoustic properties; thus, providing information about tissue and tumor composition [50]. The information can serve also to identify tumor and tissue phenotypes that are susceptible or resistant to radiation damage.

It is well-known that a tumor's biological composition is implicated in radiation response. The tumor stroma is a complex microenvironment and is constituted by a matrix of abnormal cell types, biomolecules, and immature vasculature [51,52]. Canonical radiobiology has established tumor cells as the primary targets from radiation injury; however there is also evidence to suggest that radiation damage to vascular endothelial cells mediates tumor cell death [53]. The tortuous vasculature and poor diffusion of biomolecules causes hypoxia, which is implicated in radiation resistance [54]. The disorganized microenvironment creates spatial heterogeneities which have effects on tissue microstructure and organization; thus, forming a progressive disorganized structure as tumors become more aggressive. This disorganization of the tumor is in principle detectable by QUS [55]. Early QUS studies have shown that there is a relationship between the acoustic backscatter signal and tissue properties [23,56,57]. Scatterers and nonscattering medium that constitute tissue in principle, such as cells and interstitial fluid can modulate US signals [56]. Lizzi *et al.* framework demonstrated that the scatters’ size (diameter), effective concentration, and the acoustic impedance (*CQ^2^*) contributes to properties of spectral data [56].

Indeed, in this present study, QUS imaging involved adjacent metastatic lymph nodes and the driving hypothesis was that the biological features in the infiltrated lymph nodes correlated to responses of the primary tumor and nodal disease. Previous studies, which have shown promising results, have used this hypothesis to guide their studies [58,59]. Hauser *et al.* reported pretreatment MRI-based parameters, such as the *f*-value obtained from diffusion-weighted imaging in metastatic H&N lymph nodes, were predictive of LRC and failure from XRT [58]. In that study, the mean initial *f*-value, which is representative of the vascular and perfusion signal, was greater in patients who had locoregional failure (LRF) versus those that had LRC (p = 0.01) [58]. Earlier studies using needle probes to measure oxygen have demonstrated that the pretreatment oxygen content in metastatic lymph nodes were predictive of tumor response to XRT [59,60]. In those studies, low oxygenation, as measured by the pO_2_, were reported as a statistically significant (p = 0.01) predictor of LRF [59,60]. Although this current study does not directly measure the functional and physiological parameters associated with vascularity and hypoxia, the QUS-radiomic parameters represent the tissue's biophysical structure. The results of the study here, are consistent with those previous reports, in terms of linking the pretreatment tissue parameters with predicting treatment response. The importance of evaluating metastatic lymph nodes for measuring the likelihood of LRF and as a prognostic indicator after (chemo)radiation is well-recognized [61]. Multiple regression analyses by Goguen *et al.* reported that positive lymph nodes after chemoradiation showed decreased overall survival (p < 0.001) and progression-free survival (p = 0.01) [62]. Chen *et al.* conducted a retrospective study in 117 H&N patients who received adjuvant chemoradiation [63]. There was an improvement in the 3-year local failure free survival (i.e., no evidence of recurrence) in patients with fewer metastatic lymph nodes at the time of neck dissection [63]. Results of those studies suggest that the presence of malignant lymph nodes may also represent more aggressive disease [64].

The performances of radiomic biomarkers to predict XRT response may surpass known clinical predictors, such as tumor (T) and nodal (N) extent [65]. Previous studies have shown that advanced H&N cancer that demonstrate larger tumors (T2–T4) have a greater likelihood of local failure by ≥50%, compared with early-stage disease with smaller tumors (T1) [66]. Similarly, the rate of complete XRT response in N2 disease was found to be 41%, compared with patients with N2 disease (response rate = 64%) [67]. In univariate logistic regression models, the relative risk (*RR*) of achieving a complete response to XRT was, *RR* = 10 for tumor extent (p = 0.005); whereas, using nodal involvement in the univariate model portended a *RR* = 4.2 (p = 0.037) [67]. Other predictive clinicobiological factors include increased radiosensitivity in HPV+ patients [68,69]. Significant improvements in LRC following XRT were observed in patients who expressed HPV-related markers, such as p16. The hazard ratio (HR) for LRC was HR = 0.32 (p < 0.0001) between HPV+ and HPV- patients [68].

Medical imaging will continue to play a critical role in oncology and emerging radiomic techniques will be at the forefront of advancing personalized medicine. The potential applications for QUS-based radiomic markers, when information is acquired before treatment, will be an opportunity to evaluate tumor phenotypes so that clinicians and patients can make more informed treatment decisions. The benefits of response-predictive assays include avoiding ineffective treatments and its related side effects, potentially attaining better LRC through tailored therapies and ultimately, predictive radiomic assays hold the promise of achieving better survival end points. Patients predicted up-front not to respond to two Gy fractionation to a total dose of 70 Gy could be offered altered fractionation or stereotactic radiosurgery or radiation enhancing treatments in order to improve outcomes.

The main limitations of this study include a small sample size and not all lymph nodes in the H&N were evaluated in subjects due to depth penetration. Tissue heterogeneity between lesions may have been present; for example, nine patients did not have information on HPV (p16) status as these patients received their diagnostic work from outside institutions. Future work may involve subgroup analyses for patients with HPV-, SCC since these patients have been shown to have worse survival outcomes [70]. Despite these limitations, the results here are promising and warrant further research with a greater sample sizes and with a longer follow-up period to measure patient outcomes.

## Conclusion

In this study, we present first results of QUS-radiomic markers as a response-predictive assay in H&N cancer XRT. There were statistically significant differences in QUS-radiomic parameters between complete responders versus partial responders, which included the SS-correlation, SI-contrast, SI-correlation, SI-homogeneity, and ASD-correlation. QUS-radiomic features used in multiparametric models demonstrated a high level of accuracy in classifying patients as complete responders versus partial responders after completing (chemo)XRT; thus, pretreatment QUS-radiomic features obtained from metastatic lymph nodes demonstrated an association to XRT response in the primary tumor.

## Future perspective

Radiomics-guided XRT has the promise of personalizing treatments for patients with H&N cancer. The advantage of using QUS-based biomarkers include noninvasive imaging, cost–effectiveness and portability of equipment. Imaging biomarkers from QUS can yield information that reflects the biology and physiology of tumors and is poised to provide greater clinical insight, such as measuring treatment efficacy and tumor aggressiveness. In doing so, treatments may be tailored for each patient. Artificial intelligence plays an important role in these developments; research groups are incorporating machine-learning techniques to discern optimal prediction and response models, with encouraging results. Preliminary outcomes in developing these techniques warrants further work to include larger sample groups, incorporate clinical markers such as tumor size and specifically for H&N cancer, and other predictors of XRT response such as HPV status. Future work may include exploring multimodal response models wherein biospecimen-based biomarkers are combined with imaging features. Overall, identifying response to therapy in cancer patients with the use of imaging and machine-learning techniques is a promising avenue toward precision medicine.

**Summary points**

**Background & purpose**

- Despite advances in image-guided radiotherapy (XRT) for head and neck (H&N) cancer, local relapse and death from treatment failure remains a challenge.
- Quantitative ultrasound (QUS) radiomic markers in metastatic lymph nodes of H&N cancer patients were investigated as *a priori* predictive markers for response to (chemo)XRT.

**Patients & methods**

- Thirty-two patients with diagnosed node-positive H&N cancer receiving standard-dose XRT for cure underwent QUS spectroscopy imaging of metastatic lymph nodes.
- Second-order statistical analyses using a gray-level co-occurrence matrix followed by machine-learning methods using logistic regression, naive-Bayes and k-nearest neighbor classifiers, determined best parameters for predicting patients’ clinical response.

**Results**

- There were significant differences in QUS-radiomic parameters between complete responders and partial responders, which included the spectral slope-correlation, spectral intercept (SI)-contrast, SI-correlation, SI-homogeneity and the average scatterer diameter-correlation (p < 0.05).
- The best performing univariate and bivariate features were SI-contrast (area under the curve = 0.741) and SI-con + SI-hom (area under the curve = 0.870), respectively.
- The highest accuracy (87.5%) in *a priori* treatment response prediction was achieved by the combined parameter, spectral slope-cor + SI-con + SI-hom.

**Conclusion**

- Pretreatment multivariate QUS-radiomic features obtained from metastatic lymph nodes can predict *a priori* between partial and complete response to XRT in the primary tumor.
